# Toward a Vision-Based Intelligent System: A Stacked Encoded Deep Learning Framework for Sign Language Recognition

**DOI:** 10.3390/s23229068

**Published:** 2023-11-09

**Authors:** Muhammad Islam, Mohammed Aloraini, Suliman Aladhadh, Shabana Habib, Asma Khan, Abduatif Alabdulatif, Turki M. Alanazi

**Affiliations:** 1 Department of Electrical Engineering, College of Engineering, Qassim University, Unaizah 56452, Saudi Arabia; muha.khan@qu.edu.sa; 2 Department of Information Technology, College of Computer, Qassim University, Buraydah 51452, Saudi Arabia; s.aladhadh@qu.edu.sa (S.A.); s.habibullah@qu.edu.sa (S.H.); 3 Department of Computer Science, Islamia College, Peshawar 25120, Pakistan; assmaakhan@gmail.com; 4 Department of Computer Science, College of Computer, Qassim University, Buraydah 51452, Saudi Arabia; ab.alabdulatif@qu.edu.sa; 5 Department of Electrical Engineering, College of Engineering, Jouf University, Sakaka 72388, Saudi Arabia; tmanazi@ju.edu.sa

**Keywords:** Arabic sign language recognition, convolution neural network, computer vision, deep learning, image processing, machine learning

## Abstract

Sign language recognition, an essential interface between the hearing and deaf-mute communities, faces challenges with high false positive rates and computational costs, even with the use of advanced deep learning techniques. Our proposed solution is a stacked encoded model, combining artificial intelligence (AI) with the Internet of Things (IoT), which refines feature extraction and classification to overcome these challenges. We leverage a lightweight backbone model for preliminary feature extraction and use stacked autoencoders to further refine these features. Our approach harnesses the scalability of big data, showing notable improvement in accuracy, precision, recall, F1-score, and complexity analysis. Our model’s effectiveness is demonstrated through testing on the ArSL2018 benchmark dataset, showcasing superior performance compared to state-of-the-art approaches. Additional validation through an ablation study with pre-trained convolutional neural network (CNN) models affirms our model’s efficacy across all evaluation metrics. Our work paves the way for the sustainable development of high-performing, IoT-based sign-language-recognition applications.

## 1. Introduction

About 70 million people worldwide use sign language (SL), and a machine translation system could significantly change communication between people who use SL and those who do not. Nonverbal communication that uses additional physical organs is called SL communication, which uses facial emotions, lip, hand, and eye gestures to convey information. A significant portion of daily communication for those who are hard of hearing or deaf is SL [1]. According to the World Health Organization, 5% of people on Earth have a hearing impairment. Although this number may seem tiny, it shows that over 460 million people worldwide are affected by hearing loss, 34 million of whom are children. It is predicted that more than 900 million people will have hearing loss by 2050 [2], with 1.1 billion young people at risk of becoming deaf due to noise exposure and other problems. Worldwide, hearing loss has a cost of USD 750 billion [2]. Depending on the degree of deafness, there are four types of hearing loss: mild, moderate, severe, and profound. People with severe or profound hearing loss find it challenging to communicate since they are unable to pay attention to others. A deaf person’s mental health can be significantly affected by poor communication, which can leave them feeling lonely, isolated, and unhappy. The SL used by the deaf community is gesture-based. Deaf people communicate by using gestures from SL. Interaction between a hearing person and a deaf person is complicated by the fact that the hearing person does not understand these signs. Just as spoken languages differ from each other, there are about 200 SLs around the world.

The deaf use SL, a kind of communication to exchange information. It uses gestures or signs that are major physical motions that are not part of other natural languages to convey messages. Messages are conveyed through finger and hand gestures, head nods, shoulder movements, and facial emotions. Thus, this study would allow hearing people or hearing and deaf people to talk to each other. When a hard-of-hearing or deaf person is trying to communicate something, they use gestures as a means of communication. Every symbol indicates a distinct word, letter, or feeling. Similar to how a sequence of words forms a word in spoken languages, a mixture of signals creates a sentence. SL thus has a syntax and sentence structure like a fully developed natural language. When speaking and listening in SL, facial features and lip, eye, and hand gestures are utilized to deliver meaning. SL is an important part of daily interaction with deaf people [3]. Nevertheless, it was extremely challenging for computers to comprehend hand signals due to the inconsistent size, shape, and posture of the hands or fingers in an image. SL can be tackled from two different angles: sensor-based and image-based. Users of expression frameworks do not need to employ sophisticated devices, which is their main benefit. In any case, a lot of work needs to be carried out during the preprocessing step. It is impossible to exaggerate the value of language for development. It not only serves as a channel for interpersonal communication but also helps people accept social rules and improve communication control. Even though they can hear the language spoken to them, deaf children do not learn the same terms to describe themselves as hearing children.

Recent SL research falls into two categories: Strategies based on vision and approaches based on contact. A component of the interaction technique is the interaction between users and sensing equipment. An interferometric glove is typically used to collect data on finger movement, bending, motion, and the angle of the generated sign using EMG signals, inertial measurements, or electromagnetic measurements. As input to the platform, the visual approach utilizes information from video streams taken with a camera. Additionally, it is split into presence and 3D-model-based approaches categories [4]. Most 3D model-based methods start by creating a 2D image from the position and joint angle of the hand in 3D space.

Demeanor identification uses attributes taken from a PowerPoint presentation of the image, whereas recognition relies on matching the traits [5]. Few “normal” people can understand or utilize SL, even though many hearing-impaired people have mastered it. This affects the communication of people with communication impairments and fosters a feeling of alienation between them and “normal” society. By utilizing technology that continuously transforms SL to written language and vice versa, this gap can be closed. Academics have now been helped by numerous paradigm shifts in many scientific and technological domains to suggest and put into practice SL recognition systems. Instead of using written or spoken language, people communicate with one another by using hand signals, a gesture-based method. There are 25 nations whose official language is Arabic. Only a small portion of the populace in some countries speaks Arabic [6]. Some estimates place the overall number of countries at 22 to 26. Arabic gestures are not deontological, although the language is. Jordanians, Libyans, Moroccans, Egyptians, Palestinians, and Iraqis, to name a few, are among those who speak Arabic. But every nation has a distinctive dialect. Or, to put it another way, there appear to be two dialects of Arabic: formal and informal. Arabic SL is the same across the board because they all use the same alphabet. This feature is quite helpful for research projects. A close-knit community exists among Arabs who are deaf. Low levels of interaction exist between the deaf and hearing populations, with most interactions occurring between deaf communities, deaf relatives, and occasionally playmates and professionals. Arabic SL is recognized using a continuous recognition program based on the K-nearest neighbor classifier and an Arabic SL feature-extraction method. However, Tubaiz’s method has the fundamental flaw of requiring patients to wear interferometric gloves to record data on certain activities, which in turn can be very distracting to users [7]. An interferometric glove was developed to aid in the development of a system for recognizing Arabic SL. Arabic SL can be recognized continuously using hidden Markov models (HMMs) and temporal features [8]. The goal of the study was to transcribe Arabic SL for use on portable devices. Previous work covered a wide range of SLs, but few of the studies focused on Arabic SL. Using a HMM quantifier, the researchers achieved 93% accuracy for a sample of 300 words. They used KNN and Bayesian classifications [9], which gave similar results to HMMs. This article introduces a network-matching technique for ongoing Arabic SL sentence recognition. The model makes use of decision trees and breaks down actions into stationary positions. They translate multi-word sentences with at least 63% accuracy using a polynomial runtime method. However, the above approaches, mostly based on a conventional approach to initialize weights, which involves problems of vanishing gradients and high computational complexity, achieved only a limited level of accuracy for the recognition of Arabic SL.

To address this problem, we propose a highly accurate and effective CNN-based model for Arabic SL recognition. The proposed model utilizes a lightweight EfficientNetB3 model as a backbone feature extractor; afterward, stacked autoencoders are used to refine the extracted features before the classification stage. The proposed model uses stacked coded layers and EfficientNet as the backbone architecture, which significantly increases the accuracy, decreases the false discovery rate, and enables deployment over edge devices. The main contributions of the proposed work are as follows:

- We propose an intelligent method for Arabic SL recognition that utilizes a customized variant of the EfficientNetB3 model as the foundation for feature extraction. Our model incorporates stacked autoencoders to enable robust feature selection, ensuring the optimal mapping of input images. Through extensive experimentation using various CNN models, our approach demonstrates superior recognition capabilities for Arabic sign language. The integration of densely linked coding layers further enhances the model’s performance, facilitating the accurate and efficient recognition of Arabic SL gestures.
- We conducted an extensive review of the current state-of-the-art methods for Arabic sign language recognition, with a specific focus on CNN-based approaches recognized for their high-performance capabilities in this field. Our thorough analysis revealed that the proposed model surpasses existing methods, exhibiting superior performance and holding significant potential for real-world deployment, even under limited resource constraints. By offering both efficiency and accuracy, our model presents a compelling solution for effectively and accurately recognizing Arabic sign language in various practical applications.
- The superiority of our model is substantiated through comprehensive experimentation using the ArSL2018 benchmark dataset, wherein it outperforms state-of-the-art approaches and ablation studies. Our model exhibits lower false discovery rates and achieves higher identification accuracy, affirming its exceptional performance and efficacy in Arabic sign language recognition. Furthermore, the proposed model is deployable for resource-constraint devices and can apply to different organizations.

In Section 2, various approaches and research on Arab language recognition are described in depth; Section 3 presents the proposed approach. In Section 4, the result and discussion are examined, and then the paper concludes in Section 5.

## 2. Related Work

The fourth most spoken language in the world is Arabic (Generates a Set Consulting Group 2020). In 2001, the Arab Federation of the Deaf officially declared Arabic SL as the main language for people with speech and hearing problems in Arab countries. Arabic SL is still in its infancy, even though Arabic is one of the most widely spoken languages in the world. The most general issue that Arabic SL patients realize is “diglossia”. Each country has its regional dialects that are spoken instead of written languages. As a result, the different dialects spoken have given rise to different Arabic SLs. They are as numerous as the Arab states, but all share the same alphabet and a small number of vocabulary words. Arabic is one of the more sophisticated and appealing languages and is spoken by over 380 million people around the world as the first official language. The intellectual and semantic homogeneity of Arabic is tenable [8]. The ability of NN to facilitate the recognition of Arabic SL hand gestures was the main concern of the authors in this study [10]. The main aim of this work was to illustrate the application of different types of stationary and dynamic indicators by detecting actual human movements. First, it was shown how different architectures and fully and moderately repetitive systems can be combined with a feed-forward neural network and a recurrent neural network [10]. The experimental evaluations show a 95% precision rate for the detection of stationary action, which inspired them to further explore their proposed structure. The automated detection of Arabic SL alphabets using an image-based approach was highlighted in [11]. In particular, to create an accurate sensor for the Arabic SL alphabet, several visual aspects were investigated. The extracted visible tags were fed into the One-Versus-All SVM. The results demonstrated that the Histogram of Oriented Gradients obtained promising performance, using One-Versus-All SVM and HOG identifiers. The Kinect sensor was used in [12] to develop a real-time automatic Arabic SL recognition system based on the Dynamic Time Warping coordination approach. Power and data gloves are not used by the software. Different aspects of human–computer interactions were covered in a few other studies [13]. Studies from 2011 that can identify Arabic SL with an accuracy of up to 82.22% [14,15] show that Hidden Markov models are at the center of alternative methods for SL recognition. Some other works using Hidden Markov Models can be found in [16]. A five-stage approach for an Arabic SL translator with an efficiency of 91.3% was published at the same time in [16], which focuses on the background subtraction of transcription, size, or partial invariance. Almasre and Al-Nuaim recognized 28 Arabic SL gestures using specialized detectors such as the Microsoft Kinect or Leap Motion Detectors. More recent studies have focused on understanding Arabic SL [17]. An imaging method that included the elevation, width, and intensity of the elements was used to create many CNNs and provide feedback. Instead, the frame rate of the depth footage is used by CNN to interpret the data, which also defines how vast the system is. Faster refresh rates produce more detail, while lower frame rates produce less depth. Furthermore, a new method for Arabic SL recognition was proposed in 2019 using a CNN to identify 28 letters of the Arabic language and digits from 0 to 10 [18]. In numerous training and testing permutations, the proposed seven-layer architecture was frequently taught, with the highest apparent correctness being 90.02 percent using a training dataset of 80 percent images. Finally, the researchers showed why the proposed paradigm was better than alternative strategies. Among deep neural networks, CNNs have primarily been utilized in computer-vision-based methods that generally focus on the collected images of a motion and extract its important features to identify it. Multimedia systems, emotion recognition, picture segmentation and semantic breakdown, super resolution, and other issues have all been addressed using this technology [19,20,21]. Oyedotun et al. employed a CNN and the Stacked Denoising Autoencoder to identify 24 American SL gestures [22]. Pigou et al. [23], on the other hand, recommended the use of a CNN for Italian SL recognition [24]. Another study [25] shows a remarkable CNN model that uses hand gestures to automatically recognize numbers and communicates the precise results in Bangla. This model is used in the current investigation [25]. In a related work [24,25], a CRNN module is used to estimate hand posture. Moreover, [26], recommends using a deep learning model to recognize the distinguishing features in large datasets and apply transfer learning to data collected from different individuals. In [27], a Bernoulli heat map based on deep CNN was constructed to measure head posture. Another study used separable 3D convolutional networks using a neural network to recognize dynamic hand gestures for identifying the hand signal. Another article [28] was submitted on wearable hand gesture recognition using flexible strain sensors; this is the most recent study on this topic. The authors of [29] made the most recent work-related hand gesture deformable CNN in use. Another recent effort proposed for HCI uses fingerprint detection for hand gesture recognition [30]. A small neural network is used to recognize hand gestures [31]. Learning geometric features [32] is another way to understand hand gestures. In [33], the K-nearest neighbor method provides a reliable recognition system. Arabic SL is one way to capture statistical feature extraction using a classifier. The Arabic character language is another way. Tubaiz’s method has a number of weaknesses, but the biggest one is that users have to wear instrumented gloves to capture the subtleties of a particular gesture, which is often very uncomfortable for the user. In [34], the researcher proposed using a glove with instruments to create a system for recognizing Arabic SL utilizing hidden Markov models and spatiotemporal features for the continuous recognition of Arabic SL. The authors of [35] advocated using a multiscale network for hand pose estimation. Similarly, ref. [36] investigated text translation from Arabic SL for use on portable devices. It is reported in [37] that Arabic SL can be automatically identified using sensor and picture approaches. In [38], the authors provide a programmable framework for Arabic SL hand gesture recognition using two depth cameras and two Microsoft Kinect-based machine learning algorithms. The CNN approach, which is now being used to study Arabic SL, is also unmatched [39].

In addition to the above approaches, a region-based (RCNN) is also explored for sign language recognition. For instance, various backbone pre-trained models are evaluated with RCNN, which intelligently works in numerous background scenes [40]. Next, in the case of low-resolution images, the authors of [41] used CNN for more prominent features, followed by machine learning classifiers SVM with triplet loss. Similarly, to overcome the issue of computational complexity, ref. [42] proposed a lightweight model for real-time sign language recognition, which obtained incredible performance on testing data. However, these models show better classification accuracy in the case of small datasets but limited performance over large-scale datasets. To tackle such issues, a deep CNN network was developed that was trained on massive amounts of samples and improved recognition scores [43]. This work is further enhanced in [44], where a novel deep CNN architecture is designed that obtained a tremendous semantic recognition score. In addition, to address the balancing problem, the authors of [45] developed a DL model followed by a synthetic minority oversampling technique that yielded better performance with a large number of parameters and a large model size. Therefore, it is highly desirable to develop an image-based intelligent system for Arabic hand sign recognition using novel CNN architecture.

After deep and careful analysis, we concluded that the existing work suffers from several significant limitations:

- Many approaches in the field rely on conventional weight-initialization methods, leading to issues such as vanishing gradients and high computational complexity. These challenges hinder the overall accuracy and performance of Arabic sign language recognition.
- Despite previous efforts, the existing approaches have achieved only a restricted level of accuracy in recognizing Arabic sign language. This indicates the need for further advancements to attain more precise and reliable recognition results.
- The current approaches may lack robustness when dealing with complex hand gestures, varying lighting conditions, and occlusions. This limitation hampers their effectiveness in real-world scenarios where such challenges commonly occur.
- Another notable drawback is the high computational complexity associated with the existing methods, which can impede their practical deployment, particularly in resource-constrained environments.

Addressing these limitations is crucial for advancing Arabic sign language recognition and facilitating its widespread practical application. By improving accuracy, robustness, and computational efficiency, we can enhance the effectiveness of recognition algorithms and ensure the precise interpretation of Arabic sign language gestures. Robustness enhancements will enable these systems to handle complex hand gestures, varying lighting conditions, and occlusions encountered in real-world scenarios. Additionally, reducing computational complexity will make the technology more accessible and deployable in resource-constrained environments. Overcoming these limitations will unlock the full potential of Arabic sign language recognition, promoting inclusive communication for individuals with hearing impairments.

## 3. The Proposed Model

**Figure 1:**
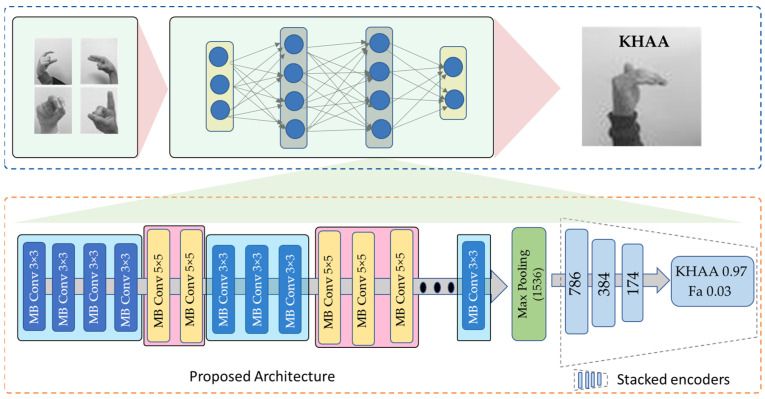
The overall architecture of the proposed model.

The literature uses a variety of techniques to identify Arabic SL. Some of these methods employ deep neural networks, which are computationally expensive and have poor accuracy. To address this problem, an efficient CNN model is proposed with less computation and that obtains acceptable performance when applied to edge devices. An overview of the proposed is shown in Figure 1. The proposed model uses EfficientNetB3 as the baseline model for feature extraction. The suggested densely connected encoder layers are used to further process the EfficientNetB3 output feature vector. Our model is briefly described in the upcoming subsections.

### 3.1. EfficientNetB3: Backbone Architecture

Several CNN-based models have been proposed in the related work for a variety of applications, including crowd estimation [43], time series prediction [46,47], classification [48], object detection [49,50], and object reidentification [51,52]. In the recent literature, several CNN designs have been created for fire recognition, including AlexNet [53], SqueezeNet [54], GoogleNet [55] MobileNet, etc. However, each CNN model has its advantages and disadvantages. To cope with this, researchers are investigating several CNN models to improve their performance by changing the width, depth, or resolution of the network through different scaling strategies. Finally, we have studied the EfficientNet design for Arabic SL recognition, in which the network dimensions are scaled using the compound scaling approach to ensure significant feature extraction from the input. The best discriminative features are then selected by sending these features through layers of tightly coupled autoencoders for feature encoding. The network then uses a SoftMax function as previously used by AlexNet [53] to perform the categorization.

### 3.2. Autoencoder

**Figure 2:**
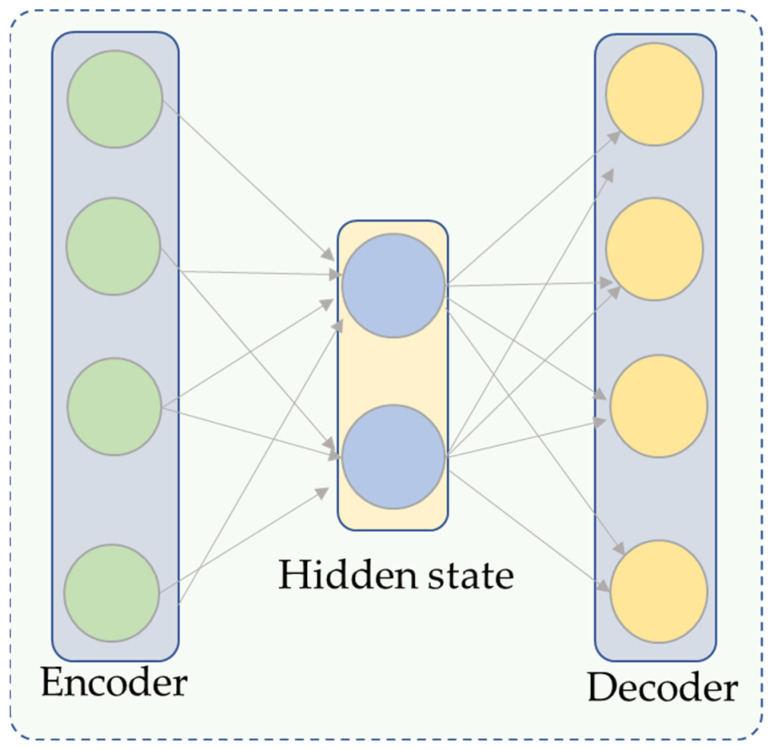
The autoencoder structure of the proposed model.

In order to comprehend uncontrolled input in a feature map, representational learning is frequently performed using autoencoder-based architectures. Input, hidden, and output layers are typically included in autoencoders. Figure 2 shows a graphical representation of these layers. The encoder maps the input into smaller dimensions, and the decoding layers then renovate it. These two components make up the bulk of an autoencoder. Consider the input data *(Xn)N* (*n* = 1), where *x_n_* is a member of the *r(m-x-l)* group, *h_n_* is the low-dimensional mapping computed from *x_n_*, and *O_n_* is the output decoder whose mathematical equations are given in Equations (1) and (2).

(1) $$h_{n}=f(w_{1}x_{n}+b_{1})$$

(2) $$O_{n}=G\left(w_{2}x_{n}+b_{2}\right),$$

where *b* is a bias term in the network, *f* is an encoding function, *G* is a decoding function, and *w* is the weight metric. The input is encoded into a compressed feature representation using the autoencoder’s encoding section. The autoencoder’s decoding component is used to reconstruct these compressed characteristics once they have been encoded. The encoding portion reduces the high-dimensional input features’ dimensions while maintaining the representation of all characteristics.

### 3.3. Weight Randomization

Three different layer types, including convolutional, pooling, and fully connected, are included in CNN. The convolutional layer extracts spatial features by using multiple layers and a dot product with weights of different filter sizes. The weights are added up in the end, and then an activation function is used to teach nonlinearity [56]. The initialization of weights and bias, which enables the extraction of distinguishing features from the input data, is the most important step in a convolutional process. At the beginning of the training, even before the weights of the first layer have been learned, the bias is optimized to minimize the error using the loss function and backpropagation. Due to the rapid changes in the gradient caused by the different values of hyperparameters such as the learning rate, vanishing gradients and bursting gradients are common problems that occur during training [57]. Therefore, researchers experimented with a variety of hyperparameters to fine-tune the model’s weights and improve performance. There are three distinct categories for weight initialization [57]. The zero initializers and the one initializer are the constant methods that are used to initialize network connections in the first category. When these initialization strategies are used, the learning algorithm’s equations often do not update the network weights, locking the model and causing each layer to have the same weights and perform similar calculations. When these distribution matrices are filled with random values, the second category’s distribution initialization approaches have a uniform or Gaussian distribution. The incorrect assignment of the necessary parameters of the network, such as the standard deviation and the mean of the distribution, can affect the training of the model and cause the problem of vanishing gradients. The third group uses the approach of initializing random weights based on past information. The model identifies local minima with inertial convergence and requires more training time since the classical CNN architecture often relies on the backpropagation error that iteratively changes the parameter [58]. Neural networks with random weight initialization have been proposed in the literature as a solution to these problems. Examples include functional link networks with random vectors. In [58], functional link networks are presented [59], and ELM is presented in [60]; Cao et al. [61] provide more information. In addition to activation functions, heuristic approaches are employed for the establishment of random layer weights. In addition to activation functions, heuristic approaches are employed for the establishment of random layer weights. Without using a process that ensures an ideal solution, the heuristics technique is employed to solve problems. Such randomization allocates the variance of the normal distribution according to the input shape. Heuristic methods lessen the problem of disappearing or expanding gradients, enabling faster convergence and reducing minima oscillation. In recent years, conventional deep learning models have shown promise in a variety of domains; however, these models still face several important challenges, such as high computational complexity, task-specific parameter adaptation, low convergence rates, etc. Compared to a standard neural network, initialization with random weights can significantly reduce the training and testing time of the model while maintaining a higher level of accuracy [60].

### 3.4. Technical Details of the Proposed Model

The following layer of the proposed model consists of densely connected coding layers based on the EfficientNetB3 design. Significant features are extracted from the input data using the EfficientNet model, and these features are then transferred to the stacked coding layers for further processing, randomly initializing the weights. The salient data are preserved, while the output of EfficientNet is compressed into these layers based on an autoencoder. We use the encoding component of the autoencoder to better capture the output characteristics of EfficientNet. Three encoding layers are included in EfficientNetB3, which produces a feature vector of 1536 dimensions, which is then compressed to a feature vector with 786 dimensions. The 786-dimensional feature vector in this example was encoded into a 384-dimensional feature vector and then into a 174-dimensional feature vector to allow the proposed model to perform at its best, as indicated in the results section. We use the technique presented [62] in stacked encoding layers with superior performance versus other models [63]. Further details are provided on densely interconnected networks by presenting a number of connectivity patterns, such as the direct connection from each layer to every other subsequent layer. The general structure of the highly connected network is shown in Figure 2. The mathematical representation of this mechanism is given in Equation (3)

(3) $$x_{L}=h_{L}(x_{0},x_{1},x_{2},\ldots x_{n-1})$$

where *L* stands for the layers, *x*_0_, *x*_1_, and *x*_2_, and *h* is the nonlinear activation function. In *x_L_*_1_, the feature map concatenations resulting from 0, 1, 2, … *L* are displayed. In this work, we concatenated the output of each encoding layer with the input of the previous layer to obtain the dimensionality with the best features for the final classification. Finally, classification is performed using the SoftMax classifier. With a learning rate of 1 × 10^−4^, a momentum of 0.9 and a loss function of binary cross-entropy, the recommended model is trained over 20 iterations. After a series of experiments, the optimizer, learning rate, and epoch count are decided upon.

## 4. Experiments and Discussions

This section describes the evaluation parameters and results obtained using the ASLR-2018 dataset, their comparison with state-of-the-art methods, and an ablation study with various deep learning models.

### 4.1. Dataset Description

**Figure 3:**
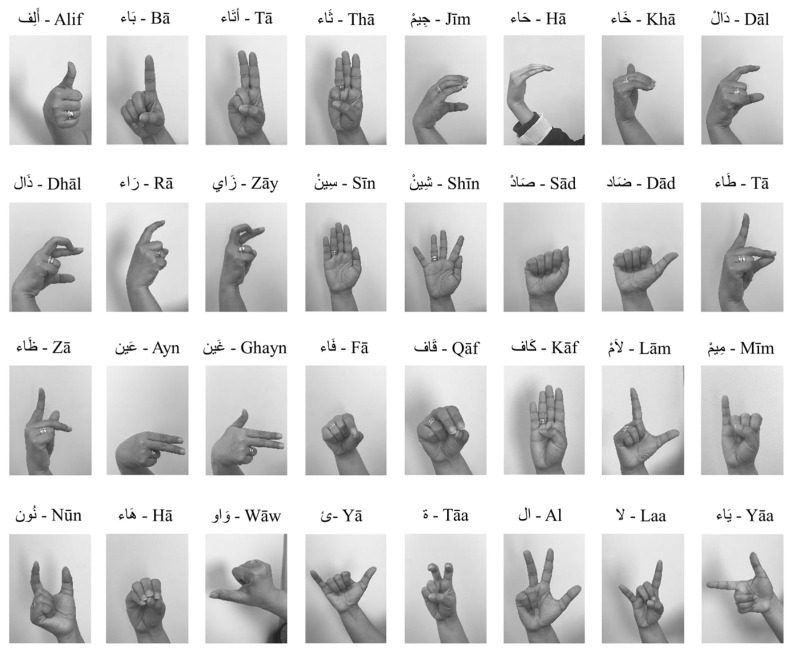
The sample images of each class in the dataset.

The dataset is accessible at ArSL2018 [42], a machine learning and deep learning conference hosted by Prince Mohammad Bin Fahd University in Al Khobar, Saudi Arabia. The collection includes 54,049 photographs of 32 typical Arabic alphabets and characters, each drawn by more than 40 different artists. Depending on the category, each class contains a different number of images. Each unique hand gesture conveys some significant information. Each class has over 1500 images, and each class’s hand gestures or signs denote a specific connotation. Visually, Figure 3 shows an example image of every subclass and its label. Thirty-two files are produced for specific storage schemes, and each folder has about 1500 images of hand movements made by people of various ages in various settings. The training and validation datasets of the model, which are explained in more detail in the next section, are treated as the directories containing these files. Data preparation is necessary in order to make the dataset more reliable and suitable for use as input in the model before discussing the model in question. The data processing is described in more detail in the following section.

### 4.2. Data Preprocessing

As was already indicated, each class has a different number of images. The model’s training performance may suffer from this disparity between the classes. To prevent this imbalance, there must be a balanced distribution of photos among all classes. The imbalance is eliminated by collecting the file names of the individual images in each class folder in a loop. From the current class folder, 1000 photos are randomly selected at each iteration, and the remaining photos are deleted. By adding together 1000 photographs from each class, 32,000 total images are then filtered. Each class has one image, each of which is 64 × 64 in size. Rescaling the images to 32 × 32 with the same dimensionality ratio will make the computations during training simple and quick.

#### 4.2.1. Data Augmentation

**Figure 4:**
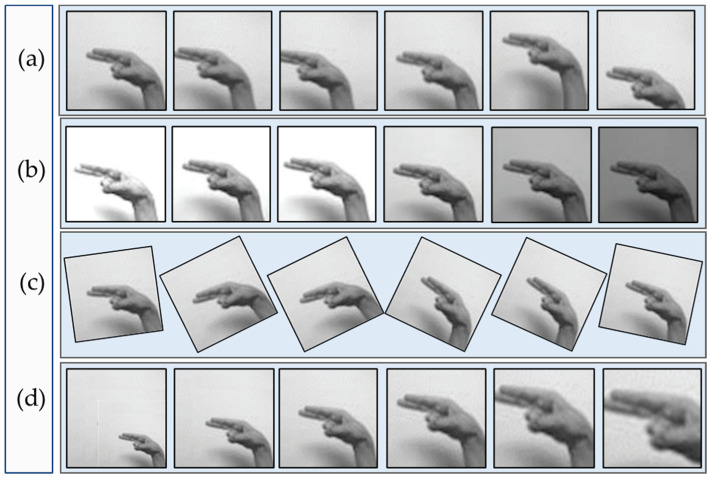
Represents different kinds of data augmentation techniques, (**a**) show the original images, (**b**) contrast adjustments, (**c**) rotations and (**d**) zooming.

The data augmentation strategy is often used to increase the size of the training dataset by creating digitally altered replicas of the original images [64]. The process leads to a more diverse and trustworthy set of images, which in turn leads to more general and informed deep learning models. By making a number of potential adjustments to the training images, the approach helps prevent the model from being overfitted or underfitted. This augmentation strategy consists of moving the object to the left, right, up, and down to a predefined boundary, and vice versa, as shown in Figure 4a. This augmentation method randomly darkens and lightens the images up to a predefined boundary, as shown in Figure 4b. Similarly, the augmentation shown in Figure 4c,d demonstrates the rotation at different degrees and zooming in or out up to the specified limit.

#### 4.2.2. Data Splitting

Several different dataset types are used to create the model’s input data. There are three different valuable datasets for computer vision projects that can be used to compare, contrast, and improve the performance of the model. These three different dataset types are used in different stages of building a machine learning model. The three different datasets are described below. A training dataset is used to train the model to determine weights or features. The model is first fitted to the training dataset, which in our case contains about 25,600 samples. In this case, the training dataset comprises 70% of the total dataset. The model is fitted to the validation dataset so that it can be objectively evaluated after training. The performance of the model is used to validate it before it is used for real-time testing on the test data set, depending on how effectively the model learns its weights. Twenty percent of the dataset is used for validation in our example of SL recognition. After the training and validation processes are finished, the performance of the proposed model is assessed, its effectiveness and accuracy are measured, and its training efficiency is evaluated using a test data set. We selected 10% of the test data from the original dataset to evaluate the performance of the model.

### 4.3. Evaluation Metric

We evaluated our model for each of the 32 sign classes for the Arabic alphabet separately using precision, recall, and Fl-score, as follows.

Precision: It is also known as positive predictive value.

(4) $$\mathrm{Precision}=\frac{\left(True\;Positive\right)}{\left(True\;Positive+False\;Positive\right)}$$

Recall: The percentage of accurate predictions divided by the total number of accurate class values is the recall percentage. The recall is described in Equation (5).

(5) $$\mathrm{Recall}=\frac{\left(True\;Positive\right)}{\left(True\;Positive+False\;Negative\right)}$$

F1-score: The F1-score is often referred to as the F-measure. The balance between recall and precision is represented by the F1-score. The F1-score only increases when both precision and recall increases. The range of F1-score values is from 0 to 1, where the higher the number, the more accurate the classification. F1-score is calculated using Equation (6).

(6) $$F1-\mathrm{score}=2\;\times \;\frac{\left(Precision\times Recall\right)}{\left(Precision+Recall\right)}$$

Accuracy is defined as the proportion of correct predictions to all correctly projected class values.

(7) $$\mathrm{Accuracy}=\frac{\left(True\;Positive+True\;Negative\right)}{\left(Ture\;Positive+True\;Negative+False\;Positive+False\;Positive\right)}$$

### 4.4. Model Evaluation

**Figure 5:**
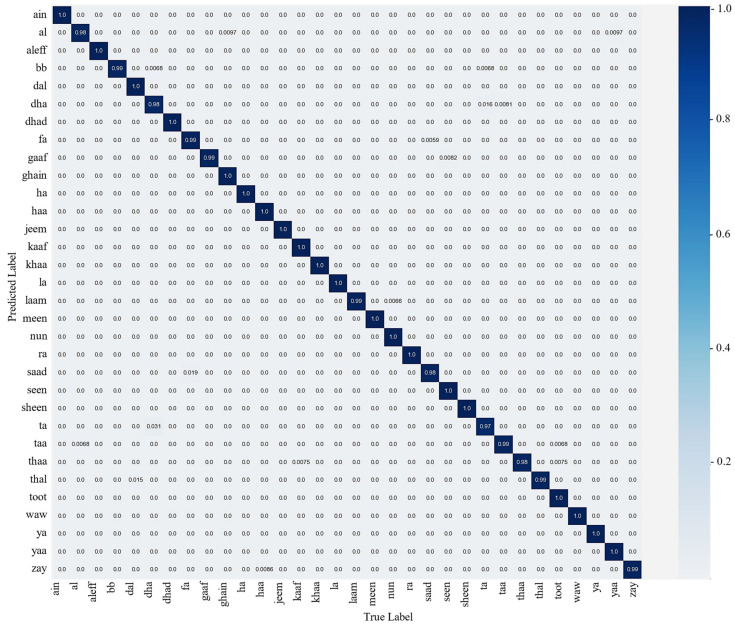
The confusion matrix of the proposed model using the test set.

This section describes the training and evaluation process of the proposed model, where the trained model is observed over testing data that are not to be examined during training. To train the proposed model, after extensive experimentation, we selected the hyperparameters, including the SGD optimizer with a momentum of 0.09, a learning rate of 0.001, a batch size of 32, and 20 epochs. The selection of these hyperparameters is dependent on the experimental data. Therefore, on the basis of the aforementioned datasets, the proposed model achieved an optimal result over 20 epochs. The result of the proposed model is examined using the confusion matrix and the classification report. The classification report represents the precision, recall, F1-score, and accuracy of the proposed model for each class, as given in Table 1, which indicates an average accuracy of 99.26%. Figure 5 illustrates the confusion matrix for each class, which shows the ratio of accurate and misclassified samples.

**Figure 6:**
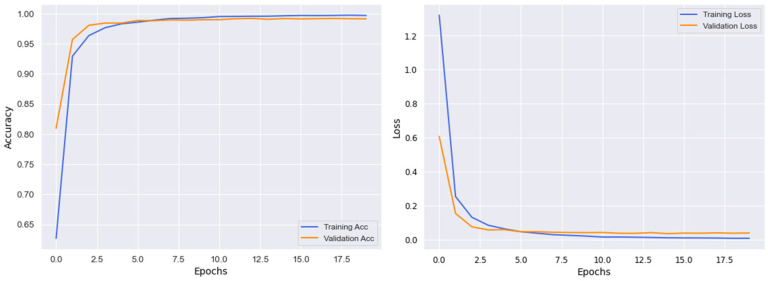
The training and validation graphs in consideration of accuracy and loss.

As explained earlier, we employed customized the EfficientNetB3 architecture for Arabic SL recognition. The reason behind choosing an EfficienetB3 is not only the better performance but also has less model complexity as compared to state-of-the-art approaches. Figure 6 shows the accuracy and loss graph for the model, and the proposed model is trained for 20 epochs. In Figure 6, the *x*-axis represents the number of epochs, and the *y*-axes show validation loss, validation accuracy, training loss, and training accuracy. In Figure 6, the training and validation accuracies increase gradually, while the training loss and validation losses decrease significantly. By achieving 100% and 99.20% training and validation accuracy, respectively, using the desired dataset, the model converges in the 10th epoch by achieving higher accuracies for both training and validation and lower loss for training and validation as well. Thus, the proposed model performs with better performance in terms of accuracy and loss, as can be observed.

### 4.5. Comparative Analysis

In this section, we compare the proposed model with several state-of-the-art models such as Alawwad et al. [40], Althagafi et al. [41], Zakariah et al. [42], Latif et al. [43], Elsayed et al. [44], Alani et al. [45], and Duwairi et al. [12] in terms of accuracy. These methods are considered state-of-the-art methods for Arabic SL recognition. In comparison, the lowest accuracy is achieved by Althagafi et al. [41], who used their model without any data augmentation approach. The second lowest is achieved by Elsayed et al. [44]. However, they used a data augmentation approach to increase the model performance. It can also be seen that Duwairi et al. [12] proposed VGGNET and obtained promising performance. Alani et al. [45] achieved higher performance of 96.59 and 97.29% accuracy with and without the data augmentation approach, respectively. However, our model performs better than the previous models, achieving 2.26% higher accuracy than Duwairi et al. [12] and 1.97% higher accuracy than Alani et al. [45]. As shown in Table 2, the proposed model achieves higher accuracy than the compared models. Furthermore, in a comparison between our model with and without the data augmentation, a notable increase of 0.91 in accuracy was observed with the use of data augmentation approach.

### 4.6. Ablation Studies

In this section, we compare the ArSL2018 performance of EFFNet with the performance of other compact deep learning models. These models include solo and integrated MobileNetV2, DenseNet121, NASNetMobile, EfficientNetB0, EfficientNetV2B0, EfficientNetV2B1, and our model with encoding layers. Table 3 shows the accuracy, recall, F1-score, and precision for each model. In Table 3, the proposed model performs well in solo baseline CNN, and the second-best performance is achieved using DensNet121, whereas the lowest performance is associated with the NasNetMobile. In the comparison, the integrated CNNs with an encoder–decoder network achieved promising results. However, our model outperformed MobileNet and previous iterations of EfficientNet in terms of all evaluation matrices. Finally, the proposed model outperformed the alternative approaches and achieved an average precision, recall, F1-score, and accuracy of 99.40%, 98.90%, 99.10%, and 99.26%, respectively. The use of pre-trained models has limited performance on SL-recognition ArSL2018 datasets, as we found through the ablation research. However, by incorporating encoder–decoder approaches int optimal feature selection, these models achieve better performance. Three feature coding layers initialized with random weights produced the best results in terms of accuracy and a decrease in false alarm rate. Thus, the proposed model provides an efficient and effective method for Arabic SL recognition. Furthermore, to compare the model size and number of parameters, the proposed model can be deployable over resource-constraint devices. In Table 3, it can be seen the MobileNetV2 model has a lower model size of 14 MB; however, the proposed model obtained higher performance than MobileNetV2 in terms of all evaluation matrices. Thus, the proposed model is the second best lightweight model in the comparison.

To comprehensively assess the impact of the modules employed in our proposed model, we employed a range of strategies, as detailed in Table 4. Initially, we examined the model’s performance when solely utilizing the encoder, achieving a 98.87% classification accuracy. Subsequently, by integrating the decoder, we observed a further enhancement in performance, yielding an accuracy of 99.03%.

The outcomes of these analyses serve to highlight the robustness of our proposed model. Notably, it not only surpassed the performance of the standalone encoder network by a substantial margin of 0.39 percentage points but also demonstrated superiority over the decoder network, surpassing it by 0.23 percentage points. These results unequivocally establish the effectiveness of our model in achieving elevated levels of performance. This underscores its adeptness at seamlessly integrating the strengths inherent in both the encoder and decoder modules, resulting in a cohesive and high-performing architecture.

## 5. Conclusions and Future Research Directions

This work aims at helping the deaf and mute community in the Arab region by developing an efficient model based on CNN coupled with staked autoencoder mechanism that converts the images of Arabic SL into letters. Various solo CNN-based and integrated stacked autoencoder models were utilized to investigate the model robustness analysis. A modified EfficientNetB3 was used to extract deeper spatial detail from the given image; afterwards, these extraction features were passed through a staked autoencoder, where the weight is randomly initialized. In the staked autoencoding network, we employed a SoftMax function after the encoding layer for the recognition of Arabic SL. The main objective of these layers is to represent the output of the model in a more abstract form, which increases the model performance in terms of precision, recall, F1-score, and accuracy. Using a solo CNN-based model, the EfficientNetB3 model achieved a higher value for accuracy of 97.20%; however, our model surpassed the EfficientNetB3 model by obtaining a 2.06% higher accuracy. Furthermore, we explored various lightweight CNN models to choose an optimal model for Arabic SL recognition in terms of precision, recall, F1-score, and accuracy. To compare with the state of the art, we dominated the results of previous Arabic SL recognition models in terms of accuracy. In comparison, the MobileNetV2 model has a lower computional complexity; for example, as the MobileNetV2 has 1.8 million fewer training parameters as compared to the proposed model. In future, we aim to use model-pronening or quantization algorithms to reduce the learning parameters and model size to increase the model’s efficiency.

## Figures and Tables

**Table 1:** The classification report of the proposed model using a test set. In addition, the support shows the number of samples.

Classes	Precision	Recall	F1-Score	Support
ain	100	100	100	174
al	100	98.05	99.06	103
aleff	100	100	100	141
bb	99.31	98.63	98.96	146
dal	98.50	1	99.24	132
dha	98.33	95.16	96.72	124
dhad	99.23	100	99.61	129
fa	100	98.82	99.40	170
gaaf	99.18	100	99.59	122
ghain	98.81	100	99.40	167
ha	100	98.33	99.15	120
haa	98.07	100	99.02	102
jeem	99.21	99.21	99.21	127
kaaf	100	100	100	135
khaa	100	100	100	89
la	100	100	100	177
laam	100	100	100	151
meem	100	100	100	140
nun	100	100	100	147
ra	100	98.48	99.23	132
saad	98.71	100	99.35	154
seen	100	100	100	132
sheen	100	100	100	124
ta	96.93	99.37	98.13	159
taa	98.00	99.32	98.65	148
thaa	100	99.24	99.62	133
thal	99.26	100	99.63	135
toot	100	99.31	99.65	145
waw	100	99.05	99.52	106
ya	100	100	100	139
yaa	100	100	100	105
zay	100	99.13	99.56	116
Average Accuracy	99.26			

**Table 2:** Comparison of the proposed model with state-of-the-art models, where WAUG and WOAUG denote the results with augmentation and the results without augmentation, respectively.

Reference	Method	WAUG	WOAUG	Accuracy (%)
Alawwad et al. [40]	Deep learning using RCNN	×	✓	93.40
Althagafi et al. [41]	Semantic segmentation CNN	✓	×	88.00
Zakariah et al. [42]	EfficientNetB4	✓	×	95.00
Latif et al. [43]	Deep learning CNN	✓	×	97.60
Elsayed et al. [44]	Deep learning CNN	✓	×	88.87
Alani et al. [45]	ArSL-CNN +SMOTE	×	✓	96.59
Alani et al. [45]	ArSL-CNN +SMOTE	✓	×	97.29
Duwairi et al. [12]	VGGNET	✓	×	97.00
The Proposed model	EfficientNetB3 with encoder and decoder network	×	✓	98.35
The Proposed model	EfficientNetB3 with encoder and decoder network	✓	×	99.26

**Table 3:** Ablation comparison of the proposed model with baseline CNN models.

Model Details	Model Size	Parameters (Millions)	Solo Baseline CNN				Baseline CNN with Encoder–Decoder Network			
Models			Precision	Recall	F1	Accuracy	Precision	Recall	F1	Accuracy
MobileNetV2	14	3.5	97.00	95.80	96.40	96.01	99.20	98.50	98.90	98.60
DenseNet121	33	8.1	98.40	96.30	97.30	97.13	99.10	98.40	98.70	98.45
NASNetMobile	23	5.3	96.00	91.10	93.10	93.00	98.20	97.80	98.00	98.00
EfficientNetB0	29	5.3	97.30	95.80	96.50	96.40	98.80	98.10	98.40	98.10
EfficientNetV2B0	29	7.2	97.40	94.30	95.60	95.50	98.50	97.70	98.10	97.90
EfficientNetV2B1	34	8.2	95.70	92.70	94.00	94.30	98.70	98.00	98.30	98.38
Our model	21	5.3	98.50	96.80	97.80	97.20	99.40	98.90	99.10	99.26

**Table 4:** Analysis of the influence of the encoder and the decoder with the proposed model.

Approach	Encoder	Decoder	Accuracy
Our model	✓	×	98.87
	×	✓	99.03
	✓	✓	99.26

## Data Availability

Data is available upon request.
